# Breastfeeding is associated with reduced risks of central obesity and hypertension in young school-aged children: a large, population-based study

**DOI:** 10.1186/s13006-023-00581-1

**Published:** 2023-09-11

**Authors:** Dan Lin, Didi Chen, Jun Huang, Yun Li, Xiaosa Wen, Ping Ou, Huijing Shi

**Affiliations:** 1 Fujian Maternity and Child Health Hospital, Fuzhou, China; 2 Department of Maternal, Child and Adolescent Health, School of Public Health, Fudan University, Shanghai, China; 3 Minhang Center of Disease Control and Prevention, Shanghai, China; 4 Minhang Branch, School of Public Health, Fudan University, Shanghai, China; 5 Minhang Maternal and Child Health Centre, Shanghai, China

**Keywords:** Breastfeeding, Body mass index, Obesity, Central obesity, Blood pressure, Childhood, Hypertension

## Abstract

**Background:**

Previous studies examined the effects of breastfeeding on measured values of body circumferences or blood pressure during childhood. However, limited data are available for the association between child feeding and a specific disease diagnosed as central obesity or hypertension. Hence, we aimed to examine whether the type and duration of breastfeeding are associated with obesity/central obesity or hypertension in young school-aged children.

**Methods:**

We matched the data obtained from a cross-sectional survey in 2019 with retrospective breastfeeding information recorded in the database. Heights, weights, waist circumferences, and blood pressures of 8480 children in first grade of primary schools in Shanghai, China were measured to diagnose obesity, central obesity, and hypertension. Data on child feeding was collected retrospectively from clinical records. Associations between the type/duration of breastfeeding and children’s measured values of body mass index, waist circumference, and blood pressure were analysed by linear regression. Associations between the type/duration of breastfeeding and risks of obesity, central obesity, and hypertension were analysed by generalised linear models.

**Results:**

Breastfeeding duration was inversely associated with blood pressure values in children in the first grade. Each month’s increase in the duration of any breastfeeding was associated with a 0.07 mmHg decrease in systolic blood pressure (*P* < 0.01) and a 0.05 mmHg decrease in diastolic blood pressure (*P* < 0.01). Any breastfeeding > one month was associated with a reduced risk of hypertension (adjusted risk ratio 0.84; 95% CI 0.73, 0.96, *P* = 0.01). Exclusive breastfeeding > one month was associated with a reduced risk of central obesity (adjusted risk ratio 0.76; 95% CI: 0.60, 0.96, *P* = 0.02). Any breastfeeding > 12 months was linked with a lower risk of hypertension (adjusted risk ratio 0.83; 95% CI 0.70, 0.98, *P* = 0.03).

**Conclusions:**

Lack of breastfeeding is associated with higher risks of central obesity and hypertension during middle childhood. As a potential component of the public health strategy to reduce population levels of metabolic and cardiovascular diseases, breastfeeding could be a vital prevention strategy.

**Supplementary Information:**

The online version contains supplementary material available at 10.1186/s13006-023-00581-1.

## Background

Globally, the number of children and adolescents with obesity has risen tenfold in the past four decades [1]. Although the rising trend in developed countries has plateaued, it has accelerated in developing countries [1]. As the largest developing country, China has the highest number of children with obesity [2]. The latest national prevalence estimates for 2015 to 2019 were 6.8% for overweight and 3.6% for obesity in children younger than six years, and 11.1% for overweight and 7.9% for obesity in children and adolescents aged 6–17 years [3]. Childhood obesity is an independent risk factor and is associated with many immediate health risks such as high blood pressure (BP), high cholesterol, impaired glucose tolerance, insulin resistance, asthma, sleep apnea, and musculoskeletal disorders [4–10]. Children affected by obesity are more likely to be obese or overweight in adulthood and suffer lifelong health problems, including cardiovascular diseases, diabetes, and cancers, which are leading causes of death [11]. China also had an increased prevalence of childhood elevated BP (EBP) and hypertension (HTN). A meta-analysis that included more than 300 000 Chinese children and adolescents reported a 10% prevalence of EBP [12], and a national survey reported that the prevalence of HTN in Chinese children and adolescents in 2015 was 19.2% [13]. Children with EBP are more likely to have HTN as adults [14–16]. EBP is also an established risk factor for cardiovascular diseases [17–19]. Although cardiovascular diseases were commonly reported in people over 50, target organ damages such as left ventricular hypertrophy or atherosclerosis originated in childhood due to EBP [20–24]. Compared with Western countries, China has higher age-adjusted mortality from cardiovascular diseases [25]. If current trends of childhood EBP continue, China will see greater increments in premature mortality from cardiovascular disease [26].

Given that obesity and EBP/HTN in children are significant public health problems in China, much attention has focused on prevention, especially during the earlier stage of life. Breastfeeding is considered a possible protective factor for obesity or higher BP during childhood or adulthood. A vast amount of literature has concentrated on the association between breastfeeding and the risk of obesity later in life [27–33]. As representative studies in recent years, the World Health Organization reported that breastfeeding reduced childhood obesity risk by up to 25% in Europe [33], and the American Academy of Pediatrics claimed a 15–30% reduction in adolescent and adult obesity [34]. However, the evidence of breastfeeding’s effect on (i) later having a healthy body mass index (BMI 18.5 to < 25) remains controversial and (ii) subsequent healthy waist circumference (WC) is very limited [35]. Furthermore, studies about breastfeeding protecting later BP were scarce. Although the experimental evidence supports that breast-milk consumption was associated with lower later BP in children born prematurely, the effects for full term children remain unclear [29, 32, 36–51]. Also, previous studies commonly reported the impacts of breastfeeding on measured values of BP but no clear disease diagnosis as EBP or HTN. At the same time, it is worth noting that nearly all the previous studies on the long-term consequences of breastfeeding and childhood obesity or BP values have been carried out primarily on Western populations living in developed nations. The limited number of studies conducted in low- and middle-income countries have not shown the same effect of breastfeeding as observed in high-income country settings [42, 52]. The situations in Asian countries are unclear and contradictory, and very few data are available from Asian populations in developing countries [43, 44, 49, 53–55].

Considering that Chinese people are more susceptible to metabolic diseases and the epidemic of cardiovascular diseases in China is growing [56–58], studies on the association between the lack of breastfeeding and later obesity/central obesity and EBP/HTN for the Chinese population are still needed. We hypothesise that the types and durations of breastfeeding are both associated with the BMI, WC, and BP values and their related health outcomes among young school-aged children. Using the data of children born and living in Shanghai, China, we tested the hypotheses: (1) there was a negative linear association between the duration of breastfeeding/exclusive breastfeeding and the BMI magnitude, WC, systolic BP (SBP), or diastolic BP (DBP) of children in their first grade in primary school, (2) there were differences among the detection rates of obesity/central obesity or EBP/HTN in first grade for children with or without breastfeeding, and (3) a type and/or duration of breastfeeding/exclusive breastfeeding was associated with a lower risk of obesity/central obesity or EBP/HTN in these young school-aged children.

## Methods

### Data sources and participants selection

All children who entered local primary schools in the 2019/2020 academic year in Minhang, Shanghai, were included in our study (*N* = 16,768). From September to December 2019, they received the first school health checks in their first semester, where anthropometric parameters and BPs were measured, and the results were recorded. We retrieved data concerning the delivery, newborn, and feeding of these children from the Health Commission-authorized database. Information about parents and births was retrieved from birth records in the hospitals. We excluded 5406 children who did not have birth data (children who were not born in this area), 2464 children who did not have postpartum visit records (children who did not grow up in this area), 126 twins, and 292 children born prematurely (gestational age was measured in weeks, from the first day of the mother’s last menstrual cycle to the day of childbirth; full term birth as at 37 gestational weeks or above [59]). Finally, the number of eligible subjects in our study was 8480 (4524 boys and 3956 girls). Oral informed consent was obtained from the parents of all children who attended the health check. Ethical approval was given by the Minhang Center for Disease Control and Prevention Ethics Committee, and the approval number is EC-2019-011. The study complied with the Ethical Principles for Medical Research Involving Human Subjects expressed in the Declaration of Helsinki.

### Anthropometric measurements and determinations of obesity and central obesity

One of the endpoints of our study is the status of overweight or obesity in children in their first grade. Data of age, sex, height, weight, and WC for the participants were collected from the school health check. The health check followed the Management Measures for Physical Examination of Primary and Secondary School Students in China, and trained nurses who participated in in-service training performed all measurements following standardised techniques to ensure validity. Bodyweight and height were measured respectively by calibrated mechanical weight scales and stadiometers, which passed the inspection of the Shanghai Compulsory Verification Center for Measuring Instruments. The weight was measured approximately to the nearest 100 g, and the height was measured approximately to the nearest 0.50 cm. The BMI was calculated as body weight in kilograms divided by height in meters squared. The WC was measured to the nearest 0.10 cm by a nonelastic flexible tape in a standing position. The tape was applied horizontally midway between the lowest rib margin and the iliac crest. The International Obesity Task Force (IOTF) defined overweight or obesity in school-aged children [60]. We defined overweight or obesity based on the age- and sex-specific BMI cutoffs under the definition. The age- and sex-specific 90th WC percentile was chosen as the cutoff to identify central obesity in children [61]. Waist-to-height ratio (WHTR) was calculated by dividing WC by height, and 0.50 was then applied as a cutoff for defining central obesity in another way in this study [62].

### BP and determination of EBP and stage 1 & 2 HTN

Data of age, sex, height, SBP, and DBP for the subjects were all collected from the results of the school health check during the children’s first semester at school. The trained nurses measured BP using an aneroid sphygmomanometer with the appropriate manual cuff for the child’s upper arm size. The children were asked to sit down and relax for at least 10 min before their BP was measured and recorded. They were requested to rest their back against the back of the chair and not cross their legs so that both of their feet were in direct contact with the floor. Their right arm was extended, flexed at the elbow, and at heart level, which had to be free of tight clothing. The results were interpreted based on Korotkoff phase I for the SBP value and phase V for the DBP value [63]. BP values were approximated to the nearest two mmHg. BP was checked twice for each child, and the time interval allowed between one measurement and another was at least five minutes. For children with normal BMI (defined by the United States Centers for Disease Control and Prevention, USCDC), SBP and DBP percentiles were calculated according to the sex-specific tables of BP levels by the Age and Height Percentile from the Clinical Practice Guideline for Screening and Management of High Blood Pressure in Children and Adolescents (2017 version) [64]. For children with overweight or obesity (defined by USCDC), SBP and DBP percentiles were calculated according to the sex-specific tables of BP levels by the Age and Height Percentile from the Fourth Report on the Diagnosis, Evaluation, and Treatment of High Blood Pressure in Children and Adolescents (2004 version) [65]. BP levels were interpreted based on each subject’s sex, age, and height (height percentile). BP values are categorised as normal (50th percentile), EBP (> 90th percentile), and HTN (≥ 95th percentile) [62, 63].

### Breastfeeding practices

Information on breastfeeding of the children was collected from pediatric clinical consultation electronic records, which were collected by healthcare professionals during post-partum follow-up visits. In this study, two exposures of interest were breastfeeding types and durations [28]. Primarily, breastfeeding types in this study included any breastfeeding (mixed or exclusive breastfeeding) and exclusive breastfeeding. Children who were ever breastfed (either mixed or exclusive breastfeeding for at least one month) were categorised into the group of “any breastfeeding”, and children who were exclusively breastfed for at least one month were categorised into the group of “exclusive breastfeeding” (exclusive breastfeeding means that the infant receives only breast milk; no other liquids or solids are given – not even water – with the exception of oral rehydration solution, or drops/syrups of vitamins, minerals or medicines) [28].

We firstly divided all the included children into two mutually exclusive feeding-practice groups of “children who were ever breastfed (any breastfeeding for one month or longer)” or “children who never breastfed or breastfed for shorter than one month (any breastfeeding shorter than one month)”. The differences in the detection rates of obesity, central obesity, and EBP/HTN and their risks in the first grade were identified. Additionally, we categorised all the children who were ever breastfed into the feeding-practice groups of “any breastfeeding for four/six months or longer” or not, or “(mixed) breastfeeding for nine/12/18/24 months or longer " or not, in order to identify the risk of obesity, central obesity and EBP/HTN in the first grade in children with different breastfeeding durations. Finally, to identify the differences in the risks of having those adverse health outcomes in the first grade in children with different exclusive breastfeeding duration, we also categorised all the children who were ever breastfed into three mutually exclusive feeding-practice groups: children who were never exclusively breastfed (mixed feeding for one month or longer, but exclusive breastfeeding for shorter than one month), children who were exclusively breastfed for shorter than six months (exclusively breastfed for one to five months), and children who were exclusively breastfed for six months.

### Other health-related variables

The health-related variables in this study included sex, birthweight (g), delivery mode (vaginal delivery or cesarean section), maternal ages at childbirth (≥ 35 years or not), and parental education levels (university-educated or not) [66]. All these data were collected from the hospital birth records.

### Statistical analysis

We tested the distribution of our continuous variables and found it is non-normal. Hence, data were presented as median (interquartile range, IQR) for continuous variables and frequencies (percentages) for categorical variables. Differences in the detection rates of adiposity, EBP, and HTN were detected by the chi-square test for categorical variables. Linear regression models were used to examine possible associations between the durations of any breastfeeding/exclusive breastfeeding and the BMI/WC/SBP/DBP values, and the regression coefficients (β)s and 95% confidence intervals (CI)s were estimated. In that model, the breastfeeding duration was examined as a continuous variable (per month increase), and the values of BMI, WC, and BP were also analysed as continuous variables (per kg/m^2^, cm, or mmHg increase).

The generalised linear models (GLM)s were applied to assess the possible associations between types and/or duration of breastfeeding and the occurrence of obesity/central obesity/EBP/HTN in children’s first year in the primary schools, and risk ratios (RR)s with their 95% CIs were estimated. In these models, the type and duration of breastfeeding was examined as non-overlapped categorical variables. Bodyweight status for these children in first grade was analysed as being obese or not, WC status was analysed as central obesity or not, and BP levels were analysed as EBP/HTN or not. Multivariate regression analyses were carried out, and these fully adjusted models included the potential confounders of sex, age at the health check, birthweight, delivery mode, maternal age, and parental education levels. For the outcomes of WC and BP, we also adjusted the BMI value obtained at the children’s first school health check. A *P*-value < 0.05 (two-sided) was considered to indicate statistical significance. We did not confine our analysis to children with complete data on all these variables, and no imputation for missing data was conducted. All statistical analyses were conducted using Stata 15.0 statistical software (StataCorp LP, College Station, TX, USA).

## Results

The characteristics of the children included in this study are shown in Table 1. The participants were born between 1 September 2010 and 31 October 2013, and the median age at the time of their school health check was 81 months of age. The median duration for any breastfeeding and exclusive breastfeeding was nine and five months, respectively. In their first grade in primary school, one in five had elevated BMI, and 6.35% were affected by obesity. More than one-fifth of them had EBP, and more than one-tenth of them had HTN.

**Table 1:** Characteristics of the children included in this study

Characteristics	Number of children	Median (Quartile 1- Quartile 3) or %
Birth and feeding		
Male	4524	53.35%
Birth weight (g)	8480	3390.00 (3110.00, 3650.00)
Cesarean delivery	4006	47.27%
Maternal age ≥ 35 years	762	9.00%
Mother university-educated	5186	66.81%
Father university-educated	5364	68.85%
Duration of any breastfeeding (months)	5355	9.00 (7.00, 11.00)
Exclusive breastfeeding duration (months)	3153	5.00 (5.00, 6.00)
**First grade in the primary school**		
Age (months)	8480	81.00 (78.00, 84.00)
Body mass index (kg/m^2^)	8476	15.68 (14.62, 17.15)
Waist circumference (cm)	8465	53.00 (50.00, 56.80)
Waist-to-height ratio	8463	0.43 (0.41, 0.46)
Systolic blood pressure (mmHg)	8461	100 (93.00, 108.00)
Diastolic blood pressure (mmHg)	8461	62 (60.00, 68.00)
Overweight or obesity	1696	20.01%
Obesity	538	6.35%
Central obesity defined by the 90th waist circumference percentile	1145	13.53%
Central obesity defined by WHTR ≥ 0.5	801	9.46%
Elevated systolic blood pressure or stage 1 & 2 hypertension	1913	22.62%
Stage 1 & 2 hypertension - systolic blood pressure	1246	14.73%
Elevated diastolic blood pressure or stage 1 & 2 hypertension	1890	22.35%
Stage 1 & 2 hypertension - diastolic blood pressure	858	10.14%

Table 2 shows the linear associations between general/exclusive breastfeeding durations and the health outcomes for the children in their first year of primary school. In the crude model, the duration of any breastfeeding (within 24 months of age) was negatively associated with the values of BMI, WC, SBP, and DBP; however, the duration of exclusive breastfeeding (within six months of age) was only negatively associated with the values of WC and SBP. In the full model, the inverse linear associations were only observed between the duration of any breastfeeding and the values of SBP and DBP. For children in their first grade, each month’s increase in the duration of any breastfeeding (within 24 months of age) was associated with a 0.07 mmHg decrease in SBP (*P* < 0.001) and a 0.05 mmHg decrease in DBP (*P* < 0.01), but no association was observed between that duration and the value of BMI or WC.

**Table 2:** Linear regression of any breastfeeding or exclusive breastfeeding duration with BMI, WC, SBP, and DBP values among children in their first grade in primary school

	Crude model			Full model	
	β (95% CI)	*P*-value		β (95% CI)	*P*-value
**Duration of any breastfeeding (in months)**					
BMI (kg/m^2^)	-0.01 (-0.02, 0.00)	0.03		-0.01 (-0.02, 0.00)	0.07
WC (cm)	-0.03 (-0.05, 0.00)	0.02		-0.01 (-0.02, 0.01)	0.49
SBP (mmHg)	-0.10 (-0.14, -0.06)	< 0.01		-0.07 (-0.11, -0.03)	< 0.01
DBP (mmHg)	-0.06 (-0.09, -0.03)	< 0.01		-0.05 (-0.08, -0.02)	< 0.01
**Exclusive breastfeeding duration (in months)**					
BMI (kg/m^2^)	-0.02 (-0.04, 0.00)	0.06		-0.02 (-0.04, 0.00)	0.09
WC (cm)	-0.08 (-0.12, -0.03)	< 0.01		-0.02 (-0.05, 0.01)	0.14
SBP (mmHg)	-0.09 (-0.17, -0.01)	0.02		0.00 (-0.08, 0.08)	0.91
DBP (mmHg)	-0.04 (-0.10, 0.01)	0.11		-0.01 (-0.07, 0.05)	0.73

CI: confidence interval; BMI: body mass index; WC: waist circumference; SBP: systolic blood pressure; DBP: diastolic blood pressure

Duration of any breastfeeding: the number of months for any breastfeeding; Exclusive breastfeeding duration: the number of months for exclusive breastfeeding

The breastfeeding duration ranged from zero to 24 months of age; the exclusive breastfeeding duration ranged from zero to six months of age

Full model adjusted for sex, age at the health check, birth weight, delivery mode, maternal age, and parental education levels. For the outcomes of WC and BP, we also adjusted the BMI value obtained at the children’s first school health check

Results in Table 3 showed differences in the detection rate for systolic and diastolic HTN among the two groups of children who were ever or never breastfed, and the detection rate in children who were never breastfed was higher (systolic HTN: 24.63% VS 21.44%; diastolic HTN: 11.18% VS 9.54%). At the same time, when children who were never breastfed were set as the referent group, we observed that children who were ever breastfed showed lower risks of having systolic EBP or HTN and diastolic HTN in their first grade (systolic EBP or HTN: adjusted RR 0.91; 95% CI 0.84, 0.99, *P* = 0.04; diastolic HTN: adjusted RR 0.84; 95% CI 0.73, 0.96, *P* = 0.01).

**Table 3:** Detection rates and risk ratios of adverse health outcomes in the first grade in primary school for children who were ever or never breastfed

Outcomes	Never breastfed	Ever breastfed	*χ*²	*P*-value	Never breastfed	Ever breastfed		
	n (%)	n (%)			Reference	Adjusted RR	95% CI	*P*-value
**Overweight or obesity**	658 (21.08)	1038 (19.39)	3.51	0.06	1.00	0.94	0.86, 1.03	0.20
**Obesity**	211 (6.76)	327 (6.11)	1.41	0.24		0.95	0.80, 1.14	0.61
**Central obesity defined by the 90th waist circumference percentile**	449 (14.40)	696 (13.01)	3.26	0.07		0.91	0.78, 1.06	0.24
**Central obesity defined by WHTR ≥ 0.5**	313 (10.04)	488 (9.13)	1.94	0.16		0.95	0.78, 1.16	0.64
**Elevated systolic blood pressure or stage 1 & 2 hypertension**	767 (24.63)	1146 (21.44)	11.41	< 0.01		0.91	0.84, 0.99	0.04
**Stage 1 & 2 hypertension - systolic blood pressure**	504 (16.18)	742 (13.88)	8.29	< 0.01		0.92	0.82, 1.03	0.15
**Elevated diastolic blood pressure or stage 1 & 2 hypertension**	732 (23.51)	1158 (21.67)	3.83	0.05		0.92	0.85, 1.01	0.07
**Stage 1 & 2 hypertension - diastolic blood pressure**	348 (11.18)	510 (9.54)	5.75	0.02		0.84	0.73, 0.96	0.01

CI: confidence interval; RR: risk ratio; WHTR: waist-to-height ratio

Full model adjusted for sex, age at the health check, birth weight, delivery mode, maternal age, and parental education levels. For the outcomes of central obesity and blood pressure, we also adjusted the BMI value obtained at the children’s first school health check

Afterwards, we set the duration of any breastfeeding as binary variables and explored their associations with obesity, central obesity, and EBP/HTN in first grade for children who were ever breastfed (Table 4). Results of multivariable regressions showed that compared with children who were breastfed for one to 11 months, children who were breastfed for 12 months and longer had lower risks of having systolic HTN in their first grade (adjusted RR 0.83; 95% CI 0.70, 0.98, *P* = 0.03). In these adjusted models, we found no association of breastfeeding durations with the outcomes of obesity.

**Table 4:** Adjusted risk ratios of adverse health outcomes in the first grade in primary school for children with different durations (months) of any breastfeeding within their first 24 months

Outcomes	Four months or longer (n = 4881)		Six months or longer (n = 4269)		Nine months or longer (n = 3071)		12 months or longer (n = 1320)		18 months or longer (n = 319)		24 months or longer (n = 83)	
	No	Yes	No	Yes	No	Yes	No	Yes	No	Yes	No	Yes
**Overweight or obesity**	1.00	0.96 (0.79, 1.18)	1.00	1.02 (0.88, 1.18)	1.00	1.04 (0.92, 1.16)	1.00	0.89 (0.78, 1.02)	1.00	0.82 (0.63, 1.07)	1.00	0.66 (0.37, 1.18)
**Obesity**		1.37 (0.88, 2.13)		1.10 (0.83, 1.46)		1.06 (0.85, 1.32)		0.91 (0.70, 1.18)		0.93 (0.57, 1.52)		0.90 (0.34, 2.33)
**Central obesity defined by the 90th waist circumference percentile**		1.43 (0.93, 2.19)		1.11 (0.88, 1.41)		1.03 (0.86, 1.24)		0.83 (0.63, 1.10)		0.98 (0.73, 1.31)		1.22 (0.73, 2.06)
**Central obesity defined by WHTR ≥ 0.5**		1.51 (0.92, 2.48)		1.19 (0.90, 1.58)		0.96 (0.77, 1.19)		0.76 (0.55, 1.06)		0.67 (0.43, 1.06)		0.73 (0.30, 1.76)
**Elevated systolic blood pressure or stage 1 & 2 hypertension**		0.98 (0.81, 1.19)		1.10 (0.96, 1.27)		0.99 (0.89, 1.10)		0.84 (0.74, 0.96)*		0.90 (0.71, 1.14)		0.85 (0.53, 1.36)
**Stage 1 & 2 hypertension - systolic blood pressure**		1.01 (0.78, 1.30)		1.13 (0.94, 1.36)		0.96 (0.84, 1.11)		0.83 (0.70, 0.98)*		0.91 (0.67, 1.24)		0.93 (0.52, 1.66)
**Elevated diastolic blood pressure or stage 1 & 2 hypertension**		1.01 (0.83, 1.22)		1.05 (0.92, 1.21)		1.04 (0.93, 1.16)		0.88 (0.78, 1.00)		0.82 (0.64, 1.05)		0.81 (0.50, 1.31)
**Stage 1 & 2 hypertension - diastolic blood pressure**		1.06 (0.78, 1.44)		1.13 (0.91, 1.41)		1.10 (0.92, 1.31)		0.96 (0.78, 1.17)		0.98 (0.68, 1.42)		0.81 (0.37, 1.75)

CI: confidence interval; RR: risk ratio; WHTR: waist-to-height ratio; *: *P*-value < 0.05

Full model adjusted for sex, age at the health check, birth weight, delivery mode, maternal age, and parental education levels. For the outcomes of central obesity and blood pressure, we also adjusted the BMI value obtained at the children’s first school health check

We also set the children who were ever breastfed but never exclusively breastfed as the referent group and estimated the risk of these adverse outcomes in the groups of children with or without the full duration of exclusive breastfeeding (six months of exclusive breastfeeding or not) (Table 5). Results showed that compared with children without exclusive breastfeeding, exclusively breastfed children had a lower risk of having central obesity defined by WHTR ≥ 0.5 in their first grade (children with an exclusive breastfeeding duration of one to five months: adjusted RR 0.76; 95% CI 0.60, 0.96, *P* = 0.02; children with six months of exclusive breastfeeding: adjusted RR 0.73; 95% CI 0.55, 0.96, *P* = 0.03). Also, compared with children without exclusive breastfeeding, children with an exclusive breastfeeding duration of one to five months had a lower risk of having central obesity defined by the 90th WC percentile in first grade (adjusted RR 0.78; 95% CI 0.64, 0.95, *P* = 0.01).

**Table 5:** Risk ratios of adverse health outcomes in the first grade in primary school for children who were ever breastfed

Outcomes	Children who were never exclusively breastfed (n = 2202)	Children who were exclusively breastfed for one to five months (n = 1658)			Children who were exclusively breastfed for six months (n = 1495)		
	Reference	Adjusted RR	95% CI	*P*-value	Adjusted RR	95% CI	*P*-value
**Overweight or obesity**	1.00	0.94	0.82, 1.08	0.41	0.94	0.82, 1.08	0.37
**Obesity**		0.86	0.66, 1.12	0.26	0.95	0.73, 1.23	0.69
**Central obesity defined by the 90th waist circumference percentile**		0.78	0.64, 0.95	0.01	0.83	0.66, 1.04	0.11
**Central obesity defined by WHTR ≥ 0.5**		0.76	0.60, 0.96	0.02	0.73	0.55, 0.96	0.03
**Elevated systolic blood pressure or stage 1 & 2 hypertension**		1.04	0.91, 1.18	0.60	1.03	0.91, 1.18	0.63
**Stage 1 & 2 hypertension - systolic blood pressure**		1.09	0.92, 1.29	0.32	1.02	0.86, 1.21	0.83
**Elevated diastolic blood pressure or stage 1 & 2 hypertension**		1.10	0.97, 1.25	0.14	1.11	0.98, 1.26	0.11
**Stage 1 & 2 hypertension - diastolic blood pressure**		1.11	0.90, 1.38	0.32	1.22	0.99, 1.50	0.07

CI: confidence interval; RR: risk ratio; WHTR: waist-to-height ratio

Full model adjusted for sex, age at the health check, birth weight, delivery mode, maternal age, and parental education levels. For the outcomes of central obesity and blood pressure, we also adjusted the BMI value obtained at the children’s first school health check

## Discussion

In this population-based study, we found that the duration of breastfeeding had a negative linear association with the BP values for children in the first grade. Our results also indicated the differences in detection rates of EBP and HTN between children who were ever or never breastfed. Conceivably, children who were never breastfed had higher detection rates, and non-breastfeeding was associated with an increased risk of having EBP or HTN in their first grade. Meanwhile, children with a breastfeeding duration of shorter than 12 months were associated with a higher risk of having systolic EBP or HTN in their first grade. Although there was no linear association between breastfeeding/exclusive breastfeeding duration and the WC value for these first graders, we found a higher risk of having WC-defined obesity during middle childhood for children without exclusive breastfeeding. However, we did not observe any association between the type or duration of breastfeeding and the BMI value or BMI-defined obesity in children in their first grade of primary school.

Setting both the breastfeeding duration during early childhood and the outcome measures in the first grade as continuous variables, previous studies have investigated the linear association between breastfeeding/exclusive breastfeeding duration and BMI, WC, and BP values in later life. For BMI values in later life, published studies showed that the duration of breastfeeding was inversely related to BMI values at age seven and even in early adulthood [67, 68]. Although no studies have challenged this negative linear association so far, our research did not support this result since the association could not be observed in our population. For WC values in later life, previous studies also found a negative dose-response relationship for the exclusive breastfeeding duration with WC in early adulthood [68, 69]. However, researchers from Australia could not confirm that association among children aged five to six, which was in accordance with our results [70]. For BP values in later life, our findings confirmed the result of a Canadian study that total breastfeeding duration was associated with the value of SBP in children aged around six years [69], and was in line with another study also from Asia that breastfeeding leads to lower BP in 7-year-old Japanese children [43]. Our results indicated that one month’s increase in the duration of any breastfeeding might reduce 0.07 mmHg of SBP value and 0.05 mmHg of DBP value. These magnitudes of the effect of longer breastfeeding duration on BP are likely to have public health implications [71]. If the causal relation exists, the small reduction in BP associated with the duration of any breastfeeding could confer important benefits on health at a population level [32]. A 1% reduction in population SBP levels is associated with a 1.5% reduction in all-cause mortality [38, 72]. Promoting a greater duration of breastfeeding is a suggested public health measure to reduce population levels of BP and BP-related health risks [29].

To the best of our knowledge, it is the first study to set EBP/HTN and central obesity as binary outcomes to explore the potential protective effects of breastfeeding against these adverse health outcomes in middle childhood. Our results indicated that breastfeeding history was associated with HTN in first grade in primary school. An American study once reported no association between breastfeeding during infancy and the risk of HTN in adulthood [73]. In that study, the authors did not observe a lower age-adjusted hazard ratio of HTN among ever-breastfed women aged 50 to 70. However, our result was not in accordance with that result. We found that compared with those who were never breastfed, the risk of having HTN in the first grade was lower for the ever-breastfed children. We also analysed the duration of any breastfeeding and suggested that children who were breastfed for 12 months and longer had a lower risk of having systolic HTN in their first grade than those with shorter breastfeeding duration. However, this result was not supported by one large cross-sectional study in China, which reported that the group of children with a duration of any breastfeeding for longer than ten months had a higher prevalence of HTN in primary school [74]. Although no clear cardiovascular event was observed for HTN in children [64, 75, 76], it tracked into adulthood and was strongly associated with increased rates of morbidity and mortality in later life [77, 78]. A meta-analysis suggested that the global prevalence of HTN in children was 4.0%, and the prevalence of HTN was 4.3% among children aged six years in 2015 [79]. In China, the prevalence of HTN was 12.4% in children and adolescents and 7.3% in children ages six to 11 from 2010 to 2011 [80]. In the present study, we found a suspected effect of breastfeeding in preventing HTN in middle childhood. As a primary prevention measure, encouraging and supporting breastfeeding has public health significance and clinical impacts on combating HTN epidemics.

Asian populations were reported to have lower BMI but higher central adiposity for given body weight when compared with matched white populations, making them more susceptible to metabolic diseases [57]. Notably, cardiometabolic risk factors are more prevalent in children with central adiposity than those with overweight or general obesity [81]. For children, central obesity is also one of the strong risk factors for metabolic disorders and cardiometabolic diseases [82], which appears to persist from childhood into adulthood [83]. In China, a national study reported that compared with children with normal BMI, the hazard ratio of being centrally obese in adulthood was more than seven times in children with central obesity [84]. Our study showed that children who were ever exclusively breastfed for any duration had a reduced risk of having central obesity in middle childhood. Previous studies did not report this possible effect of exclusive breastfeeding [85] but supported that ever breastfeeding was associated with a smaller WC during early to late childhood [69, 86]. Our result provided a new approach to preventing central obesity in children and adults before it becomes a major clinical and public health issue.

Moreover, in our population-based study, we did not observe the association between breastfeeding and the lower risk of BMI-defined overweight or obesity in children in their first grade of primary school. However, an enormous amount of research [43, 53, 87–91] confirmed the protective effect of breastfeeding on childhood obesity and supported exclusivity and a longer duration of breastfeeding can be a preventative strategy for childhood overweight and obesity. Hence, support for breastfeeding has the potential to benefit individual and public health.

### Strengths and limitations

The present study has several strengths. The majority of previous studies focused on the breastfeeding experience during infancy. The research on a large population for breastfeeding experience with 24 months was limited and unable to provide evidence for clinical and public health practice. On the contrary, our population-based study could provide stronger evidence with a large sample size and longer observation period. Moreover, the studies about breastfeeding and health indicators in later life mainly focused on linear relationships and did not involve screening or diagnostic criteria of diseases. Therefore, the relationship between breastfeeding and diseases or conditions is still vague. Our study directly investigated the associations between breastfeeding and diseases, especially for central obesity and childhood HTN, which were rarely involved by previous studies.

However, this study has its limitations. We displayed an association between breastfeeding and HTN/central obesity prevention. Nevertheless, like all previous observational studies on breastfeeding, we failed to adjust for, or poor measurement of all the possible confounding factors (maternal, child, cultural, genetic, and environmental factors) and would leave this observational study open to biased effects of breastfeeding [92]. Previous studies also reported the complex social factors for breastfeeding or childhood obesity. Economic determinants of breastfeeding for mothers include poverty, food insecurity, and employment [93, 94], and that for development of childhood obesity included poverty, food insecurity, and family stressors [95]. However, the retrospective design of the study limited our traceability to these data. Meanwhile, we obtained no information about the children’s dietary patterns, physical activity levels, and lifestyle-related behaviours, which were the most significant factors related to obesity and cardiovascular diseases. These factors we did not include and adjusted in the analysis models would greatly influence our results. Also, we looked forward to observing a “dose-response” effect that could highlight the long-term advantage of breastfeeding (for example, a longer duration of breastfeeding was associated with a lower tendency to have adverse health outcomes in first grade). Nevertheless, as the number of observation subjects was decreasing with the extension of observation time, we were unable to measure this dose-response relationship. Furthermore, BP values were only measured twice in the school health check for our subjects. It is not the diagnosis of EBP/HTN since a diagnosis should be made by trained healthcare professionals in the office setting. If a child or adolescent has auscultatory confirmed BP readings ≥ 95th percentile at three different visits, he or she was diagnosed with EBP/HTN. Meanwhile, it is impossible to identify white coat and masked HTN in these children and to confirm whether it is primary or secondary EBP/HTN, and the organic lesions cannot be excluded, which brings uncertainty to our results. In addition, as a retrospective study, we were unable to control the quality of data and information during the collection process. Hence, there could be limitations in terms of information on exclusive breastfeeding, time of breastfeeding, and mixed breastfeeding.

## Conclusions

Our study provided evidence that for early-school-age children, any breastfeeding in initial stage of life is associated with a reduced risk of HTN, and exclusive breastfeeding was associated with a reduced risk of central obesity, although the retrospective observational study design limits the causal inferences. As a potential component of the public health strategy to reduce population levels of metabolic and cardiovascular diseases, governments, societies, hospitals, communities and policies that promote breastfeeding would be helpful in improving child development and reducing health costs for individual families and at the national level.

### Electronic supplementary material

Below is the link to the electronic supplementary material.

Supplementary Material 1

Supplementary Material 2

## Data Availability

The data that underlie the results will be available for investigators after approval by Fudan University and the Minhang Center for Disease Control and Prevention. Requests for data should be please made to the corresponding author.

## Abbreviations

BMI: Body Mass Index
BP: Blood Pressure
CI: Confidence Interval
DBP: Diastolic Blood Pressure
EBP: Elevated Blood Pressure
GLM: Generalised Linear Model
HTN: Hypertension
IOTF: International Obesity Task Force
IQR: Interquartile Range
RR: Risk Ratio
USCDC: United States Centers for Disease Control and Prevention
WC: Waist Circumference
WHTR: Waist-to-height Ratio

## References

[1] NCD Risk Factor Collaboration (NCD-RisC) (2017). Worldwide trends in body-mass index, underweight, overweight, and obesity from 1975 to 2016: a pooled analysis of 2416 population-based measurement studies in 128.9 million children, adolescents, and adults. Lancet.

[2] 2015 GBD, Collaborators O, Afshin A, Forouzanfar MH, Reitsma MB, Sur P, Estep K et al. Health effects of overweight and obesity in 195 countries over 25 years. *N Engl J Med*. 2017;377(1):13–27. 10.1056/NEJMoa1614362 PMC5477817 28604169

[3] Pan XF, Wang L, Pan A (2021). Epidemiology and determinants of obesity in China. Lancet Diabetes Endocrinol.

[4] Cote AT, Harris KC, Panagiotopoulos C, Sandor GG, Devlin AM (2013). Childhood obesity and cardiovascular dysfunction. J Am Coll Cardiol.

[5] Lloyd LJ, Langley-Evans SC, McMullen S (2012). Childhood obesity and risk of the adult metabolic syndrome: a systematic review. Int J Obes (Lond).

[6] Bacha F, Gidding SS (2016). Cardiac abnormalities in youth with obesity and type 2 diabetes. Curr Diab Rep.

[7] Mohanan S, Tapp H, McWilliams A, Dulin M (2014). Obesity and asthma: pathophysiology and implications for diagnosis and management in primary care. Exp Biol Med (Maywood).

[8] Narang I, Mathew JL (2012). Childhood obesity and obstructive sleep apnea. J Nutr Metab.

[9] Pollock NK (2015). Childhood obesity, bone development, and cardiometabolic risk factors. Mol Cell Endocrinol.

[10] Africa JA, Newton KP, Schwimmer JB (2016). Lifestyle interventions including nutrition, exercise, and supplements for nonalcoholic fatty liver disease in children. Dig Dis Sci.

[11] Biro FM, Wien M (2010). Childhood obesity and adult morbidities. Am J Clin Nutr.

[12] Wang L, Song L, Liu B, Zhang L, Wu M, Cao Z (2019). Trends and status of the prevalence of elevated blood ppressure in children and adolescents in China: a systematic review and meta-analysis. Curr Hypertens Rep.

[13] Ye X, Yi Q, Shao J, Zhang Y, Zha M, Yang Q (2021). Trends in prevalence of hypertension and hypertension phenotypes among chinese children and adolescents over two decades (1991–2015). Front Cardiovasc Med.

[14] Mahoney LT, Burns TL, Stanford W, Thompson BH, Witt JD, Rost CA (1996). Coronary risk factors measured in childhood and young adult life are associated with coronary artery calcification in young adults: the Muscatine Study. J Am Coll Cardiol.

[15] Bao W, Threefoot SA, Srinivasan SR, Berenson GS (1995). Essential hypertension predicted by tracking of elevated blood pressure from childhood to adulthood: the Bogalusa Heart Study. Am J Hypertens.

[16] Stary HC (1989). Evolution and progression of atherosclerotic lesions in coronary arteries of children and young adults. Arteriosclerosis.

[17] MacMahon S, Peto R, Cutler J, Collins R, Sorlie P, Neaton J (1990). Blood pressure, stroke, and coronary heart disease. Part 1, prolonged differences in blood pressure: prospective observational studies corrected for the regression dilution bias. Lancet.

[18] Stamler J, Stamler R, Neaton JD (1993). Blood pressure, systolic and diastolic, and cardiovascular risks. US population data. Arch Intern Med.

[19] Tunstall-Pedoe H, Kuulasmaa K, Amouyel P, Arveiler D, Rajakangas AM, Pajak A (1994). Myocardial infarction and coronary deaths in the World Health Organization MONICA Project. Registration procedures, event rates, and case-fatality rates in 38 populations from 21 countries in four continents. Circulation.

[20] McCrindle BW (2010). Assessment and management of hypertension in children and adolescents. Nat Rev Cardiol.

[21] Roulet C, Bovet P, Brauchli T, Simeoni U, Xi B, Santschi V (2017). Secular trends in blood pressure in children: a systematic review. J Clin Hypertens (Greenwich).

[22] McCrindle BW, Manlhiot C, Millar K, Gibson D, Stearne K, Kilty H (2010). Population trends toward increasing cardiovascular risk factors in canadian adolescents. J Pediatr.

[23] Raitakari OT, Juonala M, Kahonen M, Taittonen L, Laitinen T, Maki-Torkko N (2003). Cardiovascular risk factors in childhood and carotid artery intima-media thickness in adulthood: the Cardiovascular Risk in Young Finns Study. JAMA.

[24] Fewtrell MS (2011). Breast-feeding and later risk of CVD and obesity: evidence from randomised trials. Proc Nutr Soc.

[25] Ohira T, Iso H (2013). Cardiovascular disease epidemiology in Asia: an overview. Circ J.

[26] Roth GA, Nguyen G, Forouzanfar MH, Mokdad AH, Naghavi M, Murray CJ (2015). Estimates of global and regional premature cardiovascular mortality in 2025. Circulation.

[27] Parikh NI, Hwang SJ, Ingelsson E, Benjamin EJ, Fox CS, Vasan RS (2009). Breastfeeding in infancy and adult cardiovascular disease risk factors. Am J Med.

[28] Victora CG, Bahl R, Barros AJ, Franca GV, Horton S, Krasevec J (2016). Breastfeeding in the 21st century: epidemiology, mechanisms, and lifelong effect. Lancet.

[29] Horta BL, Loret de Mola C, Victora CG (2015). Long-term consequences of breastfeeding on cholesterol, obesity, systolic blood pressure and type 2 diabetes: a systematic review and meta-analysis. Acta Paediatr.

[30] Ip S, Chung M, Raman G, Chew P, Magula N, DeVine D et al. Breastfeeding and maternal and infant health outcomes in developed countries. Evid Rep Technol Assess (Full Rep). 2007(153):1–186. PMC4781366 17764214

[31] Owen CG, Whincup PH, Gilg JA, Cook DG (2003). Effect of breast feeding in infancy on blood pressure in later life: systematic review and meta-analysis. BMJ.

[32] Martin RM, Gunnell D, Smith GD (2005). Breastfeeding in infancy and blood pressure in later life: systematic review and meta-analysis. Am J Epidemiol.

[33] Rito AI, Buoncristiano M, Spinelli A, Salanave B, Kunesova M, Hejgaard T (2019). Association between characteristics at birth, breastfeeding and obesity in 22 countries: the WHO european childhood obesity Surveillance Initiative - COSI 2015/2017. Obes Facts.

[34] Eidelman AI, Schanler RJ, Johnston M, Landers S, Noble L, Section on breastfeeding (2012). Breastfeeding and the use of human milk. Pediatrics.

[35] Quigley MA, Re (2006). Duration of breastfeeding and risk of overweight: a meta-analysis. Am J Epidemiol.

[36] Singhal A, Cole TJ, Lucas A (2001). Early nutrition in preterm infants and later blood pressure: two cohorts after randomised trials. Lancet.

[37] Roberts SB (2001). Prevention of hypertension in adulthood by breastfeeding?. Lancet.

[38] Martin RM, Ness AR, Gunnell D, Emmett P, Davey Smith G, Team AS (2004). Does breast-feeding in infancy lower blood pressure in childhood? The Avon Longitudinal Study of parents and children (ALSPAC). Circulation.

[39] Horta BL, Victora CG, Lima RC, Goncalves H, Guimaraes BE, Barros FC (2006). Breastfeeding duration and blood pressure among brazilian adolescents. Acta Paediatr.

[40] Kramer MS, Matush L, Vanilovich I, Platt RW, Bogdanovich N, Sevkovskaya Z (2007). Effects of prolonged and exclusive breastfeeding on child height, weight, adiposity, and blood pressure at age 6.5 y: evidence from a large randomized trial. Am J Clin Nutr.

[41] de Jonge LL, van Osch-Gevers L, Geelhoed JJ, Hofman A, Steegers EA, Helbing WA (2010). Breastfeeding is not associated with left cardiac structures and blood pressure during the first two years of life. The Generation R Study. Early Hum Dev.

[42] Brion MJ, Lawlor DA, Matijasevich A, Horta B, Anselmi L, Araujo CL (2011). What are the causal effects of breastfeeding on IQ, obesity and blood pressure? Evidence from comparing high-income with middle-income cohorts. Int J Epidemiol.

[43] Hosaka M, Asayama K, Staessen JA, Ohkubo T, Hayashi K, Tatsuta N (2013). Breastfeeding leads to lower blood pressure in 7-year-old japanese children: Tohoku Study of Child Development. Hypertens Res.

[44] Kwok MK, Leung GM, Schooling CM (2013). Breastfeeding and adolescent blood pressure: evidence from Hong Kong’s “Children of 1997” birth cohort. Am J Epidemiol.

[45] Amorim Rde J, Coelho AF, de Lira PI, Lima Mde C (2014). Is breastfeeding protective for blood pressure in schoolchildren? A cohort study in northeast Brazil. Breastfeed Med.

[46] Mora Urda AI, Pereira da Silva R, Bisi Molina Mdel C, Bresciani Salaroli L (2015). Montero Lopez Mdel P. Relationship between patterns of breastfeeding and blood pressure in brazilian and spanish schoolchildren. Nutr Hosp.

[47] Nobre LN, Lessa AD (2016). Influence of breastfeeding in the first months of life on blood pressure levels of preschool children. J Pediatr (Rio J).

[48] Martin RM, Kramer MS, Patel R, Rifas-Shiman SL, Thompson J, Yang S (2017). Effects of promoting long-term, exclusive breastfeeding on adolescent adiposity, blood pressure, and growth trajectories: a secondary analysis of a randomized clinical trial. JAMA Pediatr.

[49] Sun J, Wu L, Zhang Y, Li C, Wang Y, Mei W (2020). Association of breastfeeding duration, birth weight, and current weight status with the risk of elevated blood pressure in preschoolers. Eur J Clin Nutr.

[50] Miliku K, Moraes TJ, Becker AB, Mandhane PJ, Sears MR, Turvey SE et al. Breastfeeding in the first days of life is associated with lower blood pressure at 3 years of age. J Am Heart Assoc. 2021:e019067. 10.1161/JAHA.120.019067 PMC8475685 34284597

[51] Dong GH, Qian ZM, Trevathan E, Zeng XW, Vaughn MG, Wang J (2014). Air pollution associated hypertension and increased blood pressure may be reduced by breastfeeding in chinese children: the seven northeastern cities chinese children’s study. Int J Cardiol.

[52] Fall CH, Borja JB, Osmond C, Richter L, Bhargava SK, Martorell R (2011). Infant-feeding patterns and cardiovascular risk factors in young adulthood: data from five cohorts in low- and middle-income countries. Int J Epidemiol.

[53] Yamakawa M, Yorifuji T, Inoue S, Kato T, Doi H (2013). Breastfeeding and obesity among schoolchildren: a nationwide longitudinal survey in Japan. JAMA Pediatr.

[54] Jing H, Xu H, Wan J, Yang Y, Ding H, Chen M (2014). Effect of breastfeeding on childhood BMI and obesity: the China Family Panel Studies. Med (Baltim).

[55] Jwa SC, Fujiwara T, Kondo N (2014). Latent protective effects of breastfeeding on late childhood overweight and obesity: a nationwide prospective study. Obes (Silver Spring).

[56] Kurpad AV, Varadharajan KS, Aeberli I (2011). The thin-fat phenotype and global metabolic disease risk. Curr Opin Clin Nutr Metab Care.

[57] Ramachandran A, Chamukuttan S, Shetty SA, Arun N, Susairaj P (2012). Obesity in Asia–is it different from rest of the world. Diabetes Metab Res Rev.

[58] Gaziano TA, Bitton A, Anand S, Abrahams-Gessel S, Murphy A (2010). Growing epidemic of coronary heart disease in low- and middle-income countries. Curr Probl Cardiol.

[59] Report of a WHO Expert Committee (1995). Physical status: the use and interpretation of anthropometry. World Health Organ Tech Rep Ser.

[60] Cole TJ, Bellizzi MC, Flegal KM, Dietz WH (2000). Establishing a standard definition for child overweight and obesity worldwide: international survey. BMJ.

[61] Xi B, Zong X, Kelishadi R, Litwin M, Hong YM, Poh BK (2020). International waist circumference percentile cutoffs for central obesity in children and adolescents aged 6 to 18 years. J Clin Endocrinol Metab.

[62] McCarthy HD, Ashwell M (2006). A study of central fatness using waist-to-height ratios in UK children and adolescents over two decades supports the simple message–‘keep your waist circumference to less than half your height’. Int J Obes (Lond).

[63] Gonzalez-Jimenez E, Montero-Alonso MA, Schmidt-RioValle J, Garcia-Garcia CJ, Padez C (2015). Metabolic syndrome in spanish adolescents and its association with birth weight, breastfeeding duration, maternal smoking, and maternal obesity: a cross-sectional study. Eur J Nutr.

[64] Flynn JT, Kaelber DC, Baker-Smith CM, Blowey D, Carroll AE, Daniels SR (2017). Clinical practice guideline for screening and management of high blood pressure in children and adolescents. Pediatrics.

[65] National High Blood Pressure Education Program Working Group on High Blood Pressure in Children and Adolescents (2004). The fourth report on the diagnosis, evaluation, and treatment of high blood pressure in children and adolescents. Pediatrics.

[66] Aris IM, Rifas-Shiman SL, Li LJ, Kleinman K, Coull BA, Gold DR (2018). Pre-, perinatal, and parental predictors of body mass index trajectory milestones. J Pediatr.

[67] Vafa M, Moslehi N, Afshari S, Hossini A, Eshraghian M (2012). Relationship between breastfeeding and obesity in childhood. J Health Popul Nutr.

[68] De Kroon ML, Renders CM, Buskermolen MP, Van Wouwe JP, van Buuren S, Hirasing RA (2011). The Terneuzen Birth Cohort. Longer exclusive breastfeeding duration is associated with leaner body mass and a healthier diet in young adulthood. BMC Pediatr.

[69] Wong PD, Anderson LN, Dai DDW, Parkin PC, Maguire JL, Birken CS (2018). The association of breastfeeding duration and early childhood cardiometabolic risk. J Pediatr.

[70] Gopinath B, Subramanian I, Flood VM, Baur LA, Pfund N, Burlutsky G (2012). Relationship between breast-feeding and adiposity in infants and pre-school children. Public Health Nutr.

[71] Cope MB, Allison DB, Critical review of the World Health Organization’s (WHO) (2008). 2007 report on ‘evidence of the long-term effects of breastfeeding: systematic reviews and meta-analysis’ with respect to obesity. Obes Rev.

[72] Stamler R (1991). Implications of the INTERSALT study. Hypertension.

[73] Rich-Edwards JW, Stampfer MJ, Manson JE, Rosner B, Hu FB, Michels KB (2004). Breastfeeding during infancy and the risk of cardiovascular disease in adulthood. Epidemiology.

[74] Liang X, Xiao L, Luo Y, Xu J (2020). Prevalence and risk factors of childhood hypertension in urban-rural areas of China: a cross-sectional study. Int J Hypertens.

[75] Tran AH, Urbina EM (2020). Hypertension in children. Curr Opin Cardiol.

[76] Kochli S, Endes K, Steiner R, Engler L, Infanger D, Schmidt-Trucksass A (2019). Obesity, high blood pressure, and physical activity determine vascular phenotype in young children. Hypertension.

[77] Chen X, Wang Y (2008). Tracking of blood pressure from childhood to adulthood: a systematic review and meta-regression analysis. Circulation.

[78] Franks PW, Hanson RL, Knowler WC, Sievers ML, Bennett PH, Looker HC (2010). Childhood obesity, other cardiovascular risk factors, and premature death. N Engl J Med.

[79] Song P, Zhang Y, Yu J, Zha M, Zhu Y, Rahimi K (2019). Global prevalence of hypertension in children: a systematic review and meta-analysis. JAMA Pediatr.

[80] Yu D, Xu X, Gao X, Fang H, Ju L, Guo H (2018). Status of blood pressure and prevalence of hypertension among 6–17 years old children and adolescents in 2010–2012 in China. J Hyg Res.

[81] Mokha JS, Srinivasan SR, Dasmahapatra P, Fernandez C, Chen W, Xu J (2010). Utility of waist-to-height ratio in assessing the status of central obesity and related cardiometabolic risk profile among normal weight and overweight/obese children: the Bogalusa Heart Study. BMC Pediatr.

[82] Grigorakis DA, Georgoulis M, Psarra G, Tambalis KD, Panagiotakos DB, Sidossis LS (2016). Prevalence and lifestyle determinants of central obesity in children. Eur J Nutr.

[83] Huang TT, Ball GD, Franks PW (2007). Metabolic syndrome in youth: current issues and challenges. Appl Physiol Nutr Metab.

[84] Hou YP, Li ZX, Yang L, Zhao M, Xi B (2020). Effect of abdominal obesity in childhood on abdominal obesity in adulthood. Chin J Epidemiol.

[85] Ma J, Qiao Y, Zhao P, Li W, Katzmarzyk PT, Chaput JP (2020). Breastfeeding and childhood obesity: a 12-country study. Matern Child Nutr.

[86] Pluymen LPM, Wijga AH, Gehring U, Koppelman GH, Smit HA, van Rossem L (2019). Breastfeeding and cardiometabolic markers at age 12: a population-based birth cohort study. Int J Obes (Lond).

[87] Beyerlein A, Toschke AM, von Kries R (2008). Breastfeeding and childhood obesity: shift of the entire BMI distribution or only the upper parts?. Obes (Silver Spring).

[88] Hancox RJ, Stewart AW, Braithwaite I, Beasley R, Murphy R, Mitchell EA (2015). Association between breastfeeding and body mass index at age 6–7 years in an international survey. Pediatr Obes.

[89] Arenz S, Ruckerl R, Koletzko B, von Kries R (2004). Breast-feeding and childhood obesity–a systematic review. Int J Obes Relat Metab Disord.

[90] Qiao J, Dai LJ, Zhang Q, Ouyang YQ (2020). A meta-analysis of the association between breastfeeding and early childhood obesity. J Pediatr Nurs.

[91] Spatz DL (2014). Preventing obesity starts with breastfeeding. J Perinat Neonatal Nurs.

[92] Smithers LG, Kramer MS, Lynch JW (2015). Effects of breastfeeding on obesity and intelligence: causal insights from different study designs. JAMA Pediatr.

[93] Lesorogol C, Bond C, Dulience SJL, Iannotti L (2018). Economic determinants of breastfeeding in Haiti: the effects of poverty, food insecurity, and employment on exclusive breastfeeding in an urban population. Matern Child Nutr.

[94] Gross RS, Mendelsohn AL, Arana MM, Messito MJ. Food insecurity during pregnancy and breastfeeding by low-income hispanic mothers. Pediatrics. 2019;143(6). 10.1542/peds.2018-4113 PMC6564052 31088893

[95] Deal BJ, Huffman MD, Binns H, Stone NJ (2020). Perspective: childhood obesity requires new strategies for prevention. Adv Nutr.

