# Supplementary material for: Breastfeeding is associated with reduced risks of central obesity and hypertension in young school-aged children: a large, population-based study
Source: Int Breastfeed J. 2023 Sep 11;18:52. doi: 10.1186/s13006-023-00581-1 (PMC10496387; doi:10.1186/s13006-023-00581-1)
Supplement: Supplementary file 1 — Supplementary Material 1 [file 13006_2023_581_MOESM1_ESM.pdf]

SPRINGER NATURE

Change of authorship request form - Journals (pre-acceptance)

Important information. Please read.

- This form should be used by authors to request any change in authorship (adding/deleting authors) including changes in corresponding authors. This form should not be used for name changes. Please fully complete all sections. Use black ink and block capitals and provide each author's full name with the given name first followed by the family name.

- By signing this declaration, all authors guarantee that the order of the authors are in accordance with their scientific contribution, if applicable as different conventions apply per discipline, and that only authors have been added who made a meaningful contribution to the work.

- Please note, in author collaborations where there is formal agreement for representing the collaboration, it is sufficient for the representative or legal guarantor (usually the corresponding author) to complete and sign the Authorship Change Form on behalf of all authors, **next to the added/removed author(s)**. **(Complete Section 3, followed by Section 6.)**
  In author collaborations where there is no formal agreement for representing the collaboration and **there are more than 10 authors**, one may sign for all, provided the signer appends correspondence that attests that each of the authors have agreed to the change **and the added/removed authors sign the form**. **(Complete Section 3, followed by Section 6.)**

- Please note, we cannot investigate or mediate any authorship disputes. If you are unable to obtain agreement from all authors (including those who you wish to be removed) you must refer the matter to your institution(s) for investigation. Please inform us if you need to do this.

- If you are not able to return a fully completed form within **30 days** of the date that it was sent to the author requesting the change, we may have to withdraw your manuscript. We cannot publish manuscripts where authorship has not been agreed by all authors (including those who have been removed).

- Incomplete forms will be rejected.

- Please return/upload this form, fully completed, to the Journals Editorial Office. The Journal and/or Publisher will consider the information you have provided to decide whether to approve the proposed change in authorship. We may decide to contact your institution for more information or undertake a further investigation, if appropriate, before making a final decision.

SPRINGER NATURE

Change of authorship request form - Journals (pre-acceptance)

Section 1: Please provide the current title of manuscript

Manuscript ID no.: IBFJ-D-21-00233R3

Title: Breastfeeding is associated with reduced risks of central obesity and hypertension in young school-aged children: a large, population-based study

Section 2: Please provide the previous authorship, in the order shown on the manuscript before the changes were introduced. Please indicate the corresponding author by adding (CA) behind the name.

	First name(s)	Family name	ORCID or SCOPUS id, if available
1st author	Dan	Lin	
2nd author	Di-di	Chen	
3rd author	Jun	Huang	
4th author	Yun	Li	
5th author	Wen	Xiao-sa	
6th author	Ping	Ou	
7th author	Hua	Cao (CA)	
8th author	Huijing	Shi (CA)	
9th author			
10th author			

Please use an additional sheet if there are more than 10 authors.

SPRINGER NATURE Springer Nature is one of the world's leading global research, educational and professional publishers, created in May 2015 through the combination of Nature Publishing Group, Palgrave Macmillan, Macmillan Education and Springer Science+Business Media.

SPRINGER NATURE

Change of authorship request form - Journals (pre-acceptance)

Section 3: Please provide a justification for change. Please use this section to explain your reasons for changing the authorship of your manuscript, e.g. what necessitated the change in authorship? Please refer to the (journal) policy pages for more information about authorship. Please explain why omitted authors were not originally included and/or why authors were removed on the submitted manuscript.

The first author (Dr. Dan LIN) joined the Fujian Maternity and Child Health Hospital after graduating from Fudan University in July 2022. The publication fee will be paid by the Fujian Maternity and Child Health Hospital. But the Hospital should be listed as the author's first affiliation.
Meanwhile, in view of the contribution to the article, Dr. Cao from the Fujian Maternity and Child Health Hospital was removed from the list of authors.

Section 4: Proposed new authorship. Please provide your new authorship list in the order you would like it to appear on the manuscript. Please indicate the corresponding author by adding (CA) behind the name. If the Corresponding Author has changed, please indicate the reason under section 3.

	First name(s)	Family name (this name will appear in full on the final publication and will be searchable in various abstract and indexing databases)	Affiliated institute	E-mail address
1st author	Dan	Lin	1.Fudan University; 2.Fujian Maternity and Child Health Hospital	dlin19@fudan.edu.cn
2nd author	Di-di	Chen	1.Minhang Center of Disease Control and Prevention; 2.Fudan University	chendidi_1989@163.com
3rd author	Jun	Huang	Minhang Maternal and Child Health Centre	huangjunyian@sina.com
4th author	Yun	Li	Minhang Maternal and Child Health Centre	liyun32601@126.com
5th author	Wen	Xiao-sa	1.Minhang Center of Disease Control and Prevention; 2.Fudan University	xiaosasmmu@163.com
6th author	Ping	Ou	Fujian Maternity and Child Health Hospital	eve1019@126.com
7th author	Huijing	Shi (CA)	Fudan University	hjshi@fudan.edu.cn
8th author				
9th author				
10th author				

Please use an additional sheet if there are more than 10 authors.

SPRINGER NATURE Springer Nature is one of the world's leading global research, educational and professional publishers, created in May 2015 through the combination of Nature Publishing Group, Palgrave Macmillan, Macmillan Education and Springer Science+Business Media.

Change of authorship request form - Journals (pre-acceptance)

Section 5: Author contribution, Acknowledgement and Disclosures. Please use this section to provide a new disclosure statement and, if appropriate, acknowledge any contributors who have been removed as authors and ensure you state what contribution any new authors made (if applicable per the journal or book (series) policy). Please ensure these are updated in your manuscript - after approval of the change(s) - as our production department will not transfer the information in this form to your manuscript.

New acknowledgements:

Not applicable

New Disclosures (financial and non-financial interests, funding):

The study was supported by the Young and Middle-aged Backbone Talents Training Project of Fujian Health Commission (2022GGA033), the Special Foundation of Basic Science and Technology Resources Survey from the Ministry of Science and Technology of China (2019FY101004), and the Fudan-Minhang Health Consortium Cooperation Project from the Minhang Branch, School of Public Health, Fudan University, Shanghai (2019FM11). The funders had no role in the design of the study; in the collection, analyses, or interpretation of data; in the writing of the manuscript, or in the decision to

New Author Contributions statement (if applicable per the journal policy):

HS, DL, and DC conceptualised and designed the study, contributed to data analysis, drafted the initial manuscript, and reviewed and revised the manuscript. SW helped with study design, overviewed the study, coordinated data acquisition, and contributed to data analysis. JH and YL coordinated and supervised data collection, served as data managers at their institutions, and critically reviewed the manuscript. HS, DL and PO reviewed and revised the manuscript. All authors critically reviewed and approved the final manuscript.

State 'Not applicable' if there are no new authors.

Change of authorship request form - Journals (pre-acceptance)

Section 6: Declaration of agreement. *All* authors, unchanged, new and removed *must* sign this declaration.
(NB: Please print the form, (docu)-sign and return/upload a scanned copy. Please note that signatures that have been inserted as an image file are acceptable as long as it is handwritten. Typed names in the signature box are unacceptable.) *** Please delete as appropriate. Delete all of the bold if you were on the original authorship list and are remaining as an author.**

	First name	Family name		Signature	Date
1st author	Dan	Lin	I agree to the proposed new authorship shown in section 4 /~~**and the addition/removal*of my name to the authorship list**~~ /and the proposed change in corresponding author	林丹	2023.01.29
2nd author	Di-di	Chen	I agree to the proposed new authorship shown in section 4 /~~**and the addition/removal*of my name to the authorship list**~~ /and the proposed change in corresponding author	陈迪迪	2023.01.29
3rd author	Jun	Huang	I agree to the proposed new authorship shown in section 4 /~~**and the addition/removal*of my name to the authorship list**~~ /and the proposed change in corresponding author	黄俊	2023.01.29
4th authors	Yun	Li	I agree to the proposed new authorship shown in section 4 /~~**and the addition/removal*of my name to the authorship list**~~ /and the proposed change in corresponding author	李云	2023.01.29
5th author	Wen	Xiao-sa	I agree to the proposed new authorship shown in section 4 /~~**and the addition/removal*of my name to the authorship list**~~ /and the proposed change in corresponding author	温晓飒	2023.01.29
6th author	Ping	Ou	I agree to the proposed new authorship shown in section 4 /~~**and the addition/removal*of my name to the authorship list**~~ /and the proposed change in corresponding author	欧萍	2023.01.29
7th author	Hua	Cao	I agree to the proposed new authorship shown in section 4 /~~**and the addition/**~~**removal*of my name to the authorship list** /and the proposed change in corresponding author	曹华	2023.01.29

SPRINGER NATURE Springer Nature is one of the world's leading global research, educational and professional publishers, created in May 2015 through the combination of Nature Publishing Group, Palgrave Macmillan, Macmillan Education and Springer Science+Business Media.

SPRINGER NATURE

Change of authorship request form - Journals (pre-acceptance)

	First name	Family name		Signature	Date
8th author	Huijing	Shi	I agree to the proposed new authorship shown in section 4 /~~and the addition/removal*of my name to the authorship list~~ /and the proposed change in corresponding author	[illegible]	2023.01.29
9th author			I agree to the proposed new authorship shown in section 4 /**and the addition/removal*of my name to the authorship list** /and the proposed change in corresponding author		
10th author			I agree to the proposed new authorship shown in section 4 /**and the addition/removal*of my name to the authorship list** /and the proposed change in corresponding author		

Please use an additional sheet if there are more than 10 authors.

In case of author collaborations with formal agreement:

	Name of consortium/consortia	First name	Family name		Signature	Date
Representative/legal guarantor				I agree to the proposed new authorship shown in section 4 /**and the addition/removal*of my name to the authorship list** /and the proposed change in corresponding author		

Both added/removed authors should complete the information in the first table under Section 6.

---- End of form ----

SPRINGER NATURE Springer Nature is one of the world's leading global research, educational and professional publishers, created in May 2015 through the combination of Nature Publishing Group, Palgrave Macmillan, Macmillan Education and Springer Science+Business Media.
