# Supplementary material for: Breastfeeding is associated with reduced risks of central obesity and hypertension in young school-aged children: a large, population-based study
Source: Int Breastfeed J. 2023 Sep 11;18:52. doi: 10.1186/s13006-023-00581-1 (PMC10496387; doi:10.1186/s13006-023-00581-1)
Supplement: Supplementary file 2 — Supplementary Material 2 [file 13006_2023_581_MOESM2_ESM.docx]

**A point-by-point response to reviewers**

Reviewer reports:

Thank you for your revisions:
Response: Thank you for your careful review. Your suggestion made me learn more accurate expressions in scientific writing. Here, I would like to express my sincere gratitude to you.

Page 5, Line 11. The edit “breastfeeding normalization” doesn’t quite make sense. Consider the following edit:
“However, the evidence of breastfeeding’s effect on (i) later having a healthy body mass index (BMI 18.5 to <25) remains controversial and (ii) subsequent healthy waist circumference (WC) is very limited [35].”
Response: Thank you very much for your suggestion. After your modification, the words used here are more concise and orderly, and the meaning is more accurate. Therefore, we have modified it according to your suggestion.

Page 8, Line 38-40:
“… into two mutually exclusive feeding-practice groups of “children who were ever breastfed (any feeding for one month or longer)” or “children who were never breastfed (any feeding shorter than one month)”
A couple clarifying questions:
1. The parentheses – should this say “any breastfeeding for one month or longer” and “any breastfeeding shorter than one month”? All infants feed.
Response: Thank you for pointing out the problem for us. It is inappropriate for us to use the word“feeding”. We have changed it to “breastfeeding”.

2. Infants that had any breastfeeding for 3 weeks would be classified in the “never group”? This seems a bit inaccurate. Would it be more accurate to call this “children who never breastfed or breastfed for <1 month”

Response: Thank you for your suggestion. Your revised sentences align with the rigorous expression necessary for scientific writing. We have learned much from it and modified it according to your suggestions.
